# Socio-Demographics, Gambling Participation, Gambling Settings, and Addictive Behaviors Associated with Gambling Modes: A Population-Based Study

**DOI:** 10.1007/s10899-021-10074-7

**Published:** 2021-10-08

**Authors:** Kalle Lind, Virve Marionneau, Johanna Järvinen-Tassopoulos, Anne H. Salonen

**Affiliations:** 1 Finnish Institute for Health and Welfare, Health and Well-being Promotion Unit, P.O. Box 30, FI-00271 Helsinki, Finland; 2 Centre for Research on Addiction, Control, and Governance (CEACG), University of Helsinki, Siltavuorenpenger 1A, P.O. Box 9, 00014 Helsinki, Finland

**Keywords:** Gambling setting, Gambling mode, Gambling participation, Survey data, Population study

## Abstract

Online and land-based gambling differ in terms of participation and harms. Multimode gambling has also been distinguished as a separate mode. The current study uses the Finnish Gambling 2019 population study sample of 18–74-year-old past-year gamblers (N = 3,077) to evaluate how these gambling modes differ in terms of socio-demographics, gambling participation, gambling settings, and addictive behaviors. We used land-based gambling as the reference group in a multinomial regression model. Male gender (OR 1.48), age between 18 and 54 (OR 1.88), and high income (OR 1.87) were associated with online gambling. The odds of online gambling were higher among those who gambled at least monthly (OR 1.34) and among those with the highest gambling spending (OR 3.62). Younger age (OR 2.31), high income (OR 1.51), gambling at least four game types (OR 2.96), spending the most money on gambling (OR 4.56), and gambling in at least three gambling settings were associated with multimode gambling. Socio-demographics and gambling participation were indicators of gambling modes. Online gambling was more intensive while multimode gambling was more frequent and versatile than land-based gambling. However, this was not reflected as increased addictive behaviors, probably due to the harmful nature of Finnish land-based gambling.

## Introduction

Gambling participation and gambling-related harms vary depending on population groups and gambling products. Previous research has shown that men and younger adults tend to gamble proportionately more, and experience more harms than women and older adults (Raybould et al. 2021; Castrén et al. 2018; Abbott et al. 2018), although there have also been signs of narrowing gender differences (Castrén et al. 2018). Studies have also observed differences in participation and levels of harm between occupational and socio-economic groups (Raybould et al. 2021; Binde and Romild 2020). Some gambling products are also linked to more harm than others. Fast games, such as electronic gambling machines (EGMs) in both online and land-based environments, other casino games, and scratch cards have been connected to elevated harm levels (Raybould et al. 2021; Gainsbury et al. 2019; Salonen et al. 2020a; Castrén et al. 2017). Notably EGMs have been shown to both attract those already experiencing problems with their gambling, but also creating gambling-related harms and problems (Livingstone et al. 2019; Dowling et al. 2005).

Gambling participation and harms have also been connected to channels or modes of access. A recent systematic review (Raybould et al. 2021) found that overall, online gambling appears to be connected to an increase in harms in comparison to land-based gambling, although this may also be related to higher gambling involvement rather than the gambling mode (Baggio et al. 2017). The socio-demographic profile of online gamblers has also been shown to differ from that of land-based gamblers (Mora-Salgueiro et al. 2021). Even the same activity provided in online modalities may lead to more harms than in land-based environments (Gainsbury et al. 2019; Gainsbury 2015).

Previous studies have used varying definitions of online gambling, ranging from lifetime prevalence to weekly participation (Mora-Salgueiro et al. 2021). Most studies comparing land-based and online gambling have also not considered multimode gamblers, or these have been included into the category of online gamblers. Multimode gambling (also known as mixed-mode gambling) is here defined as gambling that takes place both online and in land-based environments. Multimode gambling appears to constitute a separate type of modality both in terms of gambling participation and related harms (Wardle et al. 2011; González-Roz et al. 2017). Furthermore, the more intensive gambling involvement in multimode gambling may increase the number of gambling products played and thereby the risks of higher gambling involvement and gambling on more harmful products.

The aim of the current study is to use the Finnish Gambling 2019 population study to address the question of how land-based, online, and multimode gambling differ in terms of socio-demographics, gambling participation, gambling settings, and addictive behaviors.

## Characteristics of Multimode Gambling

### Age Groups

Online gambling may appear as a particularly attractive activity for young people owing to the increased accessibility of online gambling in the last decade and new products such as e-sports (King et al. 2020). A recent review (King et al. 2020) suggest that, internationally, approximately 5–15 % of underage gamblers (12–17-year-olds) can access online gambling environments. Existing evidence, although limited, also shows that multimode gamblers may be younger than online or land-based gamblers (Gainsbury et al. 2015). In a Spanish sample of 1,313 adolescents (14–18 years old), 34.7 % had gambled land-based only, 0.6 % online only and 3.9 % were multimode gamblers (González-Roz et al. 2017). The British Gambling Prevalence Survey (Wardle et al. 2011) found that online gambling was the most common among 35–54-year-olds. Multimode gambling was the most common among 16–34-year-olds who gambled on different activities in online and land-based environments, and among 16-34-year-olds and 35–54-year-olds gamblers who participated in the same activities in online and land-based environments.

### Gender

Multimode gamblers are also more likely to be males than females (Wardle et al. 2011; Salonen et al. 2018). In the Finnish population study from 2016, the share of women (25 %) gambling online was higher than that of men (8 %), but the proportion of men was higher among multimode gamblers (65 % male, 41 % female) (Salonen et al. 2018). Male gambling has been associated with public spaces and female gambling with home settings which may explain why in some samples online gambling has been more typical of females (Svensson et al. 2011).

### Socio-Economic Status

The socio-economic situation and education levels differ between multimode and online or land-based gamblers. The British Gambling Prevalence Survey looked at gambling modes by personal income tertiles (Wardle et al. 2011). The results showed that the lowest and highest earning tertiles gambled more in land-based environments. The highest earning tertile also gambled more in online and multimode environments in comparison to the two other groups. The impact of socio-economic status may also differ based on gender. In the Finnish Gambling 2015 population study (Edgren et al. 2017), online and land-based gamblers were compared by employment status. Results showed that the highest odds ratio (OR) for online gambling was for males in fulltime employment and for females who were either students or homemakers. Another factor that may impact differences in the socio-economic profiles of gambling modes is related to the types of gambling products.

### Gambling Problems and Harms

In addition to differing player profiles, multimode gambling may be related to different levels of gambling harms than online or land-based participation. Land-based and online gamblers are less involved in gambling than multimode players in terms of the proportion of regular gamblers and the number of gambling products undertaken (Wardle et al. 2011). Multimode gamblers also have higher problem gambling prevalence rates (Wardle et al. 2011; Gainsbury et al. 2015; Blaszczynski et al. 2016). In the Spanish adolescent survey described above (González-Roz et al. 2017), 10.2 % of multimode gamblers were classified as problem gamblers, as opposed to 2.3 % of land-based gamblers. The higher rates of gambling problems and gambling-related harm in multimode gambling may be related to higher participation or the types of gambling products that are played: In Australia (Gainsbury et al. 2015; Blaszczynski et al. 2016), multimode gambling has been connected to a larger variety of gambling forms in comparison to land-based or online gambling.

### Other Addictive Behaviors

Multimode gambling may also be linked to other addictive behaviors, such as alcohol consumption or smoking. Particularly alcohol consumption may be influenced by specific events such as jackpot wins, bonus features, or winning streaks in land-based conditions (cf. playing EGMs in casinos) (Tobias-Webb et al. 2019), but alcohol consumption may also be linked to online gambling. In Finnish population study (Edgren et al. 2017), online gamblers (including multimode gamblers) were more typically smokers and had risky alcohol consumption than land-based gamblers. Similarly, in Australia, multimode gambling has been connected to higher alcohol consumption than online gambling (Blaszczynski et al. 2016).

## Methods and Data

### Data

The Finnish Gambling 2019 population study examines gambling and problem gambling among 15–74-year-old Finns living in Mainland Finland. The study was conducted by the Finnish Institute for Health and Welfare and commissioned and financed by the Ministry of Social Affairs and Health (Sect. 52 of the Lotteries Act). The sample was selected by means of systematic random sampling from a sampling frame formed on the basis of the register-based Population Register Centre database. Persons living in institutions (prisoners, infirm, etc.) and persons whose mother tongue was other than Finnish, Swedish or Sámi were excluded from the study. The study was carried out in Finnish or Swedish (the two official languages of Finland).

The data were collected by Statistics Finland using computer-assisted telephone interviews (CATI) between September 2nd and December 13th, in 2019. A cover letter and a brochure on the study were sent to 7,800 potential participants. Different types of cover letters were used: a regular one and a letter for those who were hesitant to respond. A separate same content brochure with a more youthful appearance was also produced for 15–30-year-olds. A more detailed description of the data collection is available in English in the statistical report (Salonen et al. 2020a). On average, interviews lasted for 24 min. Altogether, 3,994 interviews were acceptably completed. After reducing over-coverage, the response rate was 51.9 %. Although slightly lower, the response rate is comparable to other international studies. In similar CATI-based gambling-studies the response rate has been 52.5 % on average (Williams et al. 2012).

For the purpose of this article, only data on 18–74-year-old past-year gamblers were used (N = 3,077). 15–17-year-olds were excluded because the age limit for gambling in Finland is 18 years. Previous studies have also shown that underaged gambling mainly occur in land-based contexts in Finland (Salonen et al. 2020b). Converted to a population estimate, the included proportion corresponds to 2,917,000 gamblers living in Mainland Finland. This represents an important part of the population: Overall, there were 3,722,323 people aged 15 to 74 years living in Mainland Finland in 2019.

### Context

The Finnish case is interesting from the perspective of multimode gambling because the shares of online and land-based gambling in the country are similar. Gambling in Mainland Finland is offered by a state-owned monopoly provider, Veikkaus. Veikkaus products are available online and in land-based gambling locations, including arcades, the Helsinki casino, and convenience locations, such as kiosks, grocery stores, restaurants, and gas stations. Foreign-based online sites are not currently blocked or legally prohibited, but also not officially licensed. Additional EGMs and casino games are provided on passenger cruise ships sailing between Helsinki (Finland), Mariehamn (Åland islands), Stockholm (Sweden) and Tallinn (Estonia). These are operated by Ålands Penningautomatförening (PAF), a monopolistic operator that operates onboard cruise ships under the legislation of the Finnish autonomous Åland Islands. PAF also offers unlicensed online gambling to Mainland Finland. In 2020, Veikkaus controlled 80 % of the Finnish gambling market and 62.7 % of the online market. The online channel makes up 43 % of Veikkaus gross gambling revenue while 57 % is made up of the land-based channel (Veikkaus 2021 based on the estimations of H2 Gambling Capital).

### The Gambling Modes

In this paper, gambling modes are examined from the perspective of three distinct gambler groups: online only gamblers, land-based only gamblers, and multimode gamblers. In the interest of brevity, we refer to land-based only and online only gambling here simply as land-based and online gambling. The three modes are determined by gambling participation within the previous 12 months. The term ‘land-based’ gambling refers to games offered in environments other than the internet, for example at operator’s gambling locations and distributor locations. Online gambling involves gambling using either a computer or a mobile device. The gambling mode variable was recoded using several different items. In the survey the respondents were offered options of different game types to choose from: whether they had engaged in them or not and whether they played the online or the land-based version. If the game type was available only land-based or online, this information was used to define the gambling mode. The survey also included a separate question concerning gambling mode in general, assuring that no gamblers went unidentified. In the current study, we have not separated between modes of access to online gambling (e.g., mobile and computer), although mobile users may be at a higher risk for gambling-related problems (Gainsbury et al. 2015).

### Socio-Demographics

Information about the gender and age of respondents were retrieved from the administrative registers of Statistics Finland and combined with the survey data. The study population was divided into six age groups (Table 1). Furthermore, information about the employment status and personal net income were inquired. Personal net income was recoded based on the quartiles (Q1–Q4).

### Gambling Participation

To examine the number of game types played, a list of game types was provided with a “yes/no” option for responses. In the analysis, games provided in the monopoly platform were recoded into nine game types: weekly lottery games, daily lottery games, EGMs outside casino, scratch games, betting games, horse games, online poker, casino games operated by a croupier outside the casino, and games in casino Helsinki. In addition, PAF games on cruise ships, PAF online games, and other offshore gambling were combined and treated as one game type. Private betting was considered a further separate game type.

Based on these 11 game types, we formed a classification of gambling versatility based on whether respondents had gambled on one game type, two game types, three game types, or four or more game types. For each game type, the frequency of past-year gambling was inquired about using the following options: daily or several times a week, once a week, 1–3 times a month or rarely. Overall gambling frequency was then defined according to the game in which the respondent was the most active.

Furthermore, those who had participated in any past-year gambling were asked to estimate the amount they had spent on gambling. They were allowed to report their gambling expenditure (in €) based on the gambling frequency of their choice.

Gambling settings were inquired about by asking, “What sort of environments did you gamble in during the past 12 months?” with separate list of choices provided. These choices were recoded as follows: domestic space (home), working space (own work), daily life space (grocery store, kiosk, gas station), leisure space onshore (café, restaurant, but excluding cruise ships), gambling venue (casino, gambling arcade), and other. A new variable was created to reflect the number of gambling settings chosen.

### Variables Measuring Addictive Behaviors

Gambling severity and risky alcohol consumption were used as measures of addictive behavior. Problem gambling was measured using the South Oaks Gambling Screen including 20 items (SOGS; Lesieur and Blume 1987; 1993). SOGS has been used as the primary instrument for assessing the prevalence of problem gambling in Finland (Salonen et al. 2020a). An Alcohol Use Disorders Identification Test (Audit-C) was used to assess risky alcohol consumption. Based on the Finnish Care Guidelines, the score of five or more among women and six or more among men was used as a criterion for past-year risk-level alcohol consumption with the Audit-C (Käypä hoito 2018). The Cronbach’s alpha value was 0.76 for the SOGS was 0.76 and 0.70 for the Audit-C.

### Data Analyses

Information on the age, gender, and region of residence were used to calibrate the data weights. This process aimed at reducing the bias caused by non-response and at improving the efficiency of estimation. The analyses were performed with SPSS (SPSS version 27.0, Chicago, IL, USA). With the descriptive analyses, statistical significance was determined by Pearson’s chi-squared tests. Furthermore, multivariate-adjusted multinomial logistic regression analysis was used to examine the correlates reflecting socio-demographics, gambling participation, and addictive behavior, as well as their association with gambling modes. Selected factors carry theoretical evidence from previous studies, and they were dichotomized and added simultaneously into the models. To precisely optimize the models, the number of gambling settings was included as a gambling-related factor. Multicollinearity was tested by applying the variance inflation factor (VIF) and the results indicate that our predictors are not correlated (sub 5 values). Also correlation matrix suggests that the predictors are not strongly correlated (no absolute correlation coefficients of > 0.7 among two or more predictors). Furthermore, evidence from previous studies and information based on Table 1 were used while defining the reference categories in the models.

### Ethics

The research protocol was approved by The Ethics Committee of the Finnish Institute for Health and Welfare (Statement THL/774/6.02.01/2019). Potential participants were informed about the principles of voluntary participation. The privacy notice for scientific research was published based the General Data Protection Regulation (GDPR) and can be found on the study website (www.thl.fi/rahapelitutkimus2019). The research data, excluding register data linked to them, are available and openly accessible for research purposes from the Finnish Social Science Data Archive (FSD).

## Results

Of the three gambling modes, land-based gambling (52.6 %) was the most common, followed by multimode gambling (29.1 %). Online gambling was the least common mode (18.3 %) (see Table 1).

### Descriptive Analysis

Based on the descriptive analysis, socio-demographics, gambling behaviors, and addictive behaviors were significantly associated with past-year gambling modes (Table 1). The proportion of land-based gamblers was higher among women (63.1 %) than among men (43.1 %) while the situation was the opposite with online and multimode gambling (Table 1). Every fifth (21.7 %) woman and every third man (35.8 %) was a multimode gambler. Land-based gambling was the most common mode among the oldest age groups: 73.3 % of 65–74-year-olds and 57.0 % of 55–64-year-olds had only gambled in land-based environments. Respectively, multimode gambling was more common among the young, particularly 25–34-year-old (41.7 %), respondents. In addition, land-based gambling was the most prevalent in the lower income quartiles (Q1: 66.5 %; Q2: 60.7 %). At the same time, online gambling and multimode gambling were the most prevalent in the higher income quartiles.

**Table 1:** Socio-demographics, gambling participation and addictive behaviours associated with past-year gambling mode

	Land-based gambling % (n)	Online gambling % (n)	Multimode gambling % (n)	p
	52.6 (1659)	18.3 (566)	29.1 (852)	
*Gender*				< 0.001
Woman	63.1 (917)	15.2 (224)	21.7 (294)	
Man	43.1 (742)	21.1 (342)	35.8 (558)	
*Age group*				< 0.001
18–24 years	48.6 (102)	12.4 (27)	39.0 (86)	
25–34 years	44.2 (206)	14.1 (67)	41.7 (197)	
35–44 years	41.9 (203)	21.6 (107)	36.5 (182)	
45–54 years	47.7 (267)	22.0 (125)	30.3 (169)	
55–64 years	57.0 (364)	21.3 (134)	21.8 (138)	
65–74 years	73.3 (517)	15.4 (106)	11.3 (80)	
*Net income in €*				< 0.001
Q1 (Low) or missing	66.5 (356)	12.1 (58)	21.4 (99)	
Q2 (Average)	60.7 (453)	14.6 (108)	24.8 (172)	
Q3 (Average)	45.9 (415)	18.9 (170)	35.2 (297)	
Q4 (High)	45.3 (435)	24.0 (230)	30.7 (284)	
*Gambling frequency*				< 0.001
Rarely	69.0 (789)	13.5 (154)	17.5 (190)	
1–3 times/month	41.3 (312)	19.9 (141)	38.9 (262)	
Once a week	46.6 (458)	23.3 (215)	30.1 (264)	
Daily or several times/week	31.9 (100)	18.8 (56)	49.3 (136)	
*Number of game types*				< 0.001
1 game type	72.5 (616)	22.0 (187)	5.5 (46)	
2 game types	63.7 (548)	19.2 (162)	17.1 (136)	
3 game types	48.1 (291)	18.5 (108)	33.3 (193)	
≥ 4 game types	24.8 (204)	13.4 (109)	61.7 (477)	
*Gambling expenditure*				< 0.001
Q1 (Low)	80.9 (611)	10.2 (77)	8.9 (63)	
Q2	49.4 (377)	20.7 (149)	29.9 (205)	
Q3	42.8 (359)	24.4 (192)	32.8 (243)	
Q4 (High)	36.5 (312)	18.3 (148)	45.2 (341)	
*Number of gambling spaces/places*				< 0.001
None or missing	80.0 (36)	17.8 (7)	-	
One space	70.8 (1177)	24.5 (405)	4.7 (74)	
Two spaces	41.9 (360)	13.6 (109)	44.5 (372)	
Three or more spaces	16.1 (86)	8.1 (45)	75.7 (405)	
*Gambling severity* ^a^				< 0.001
SOGS = 0	55.6 (1443)	18.8 (483)	25.6 (623)	
SOGS = 1–2	41.0 (176)	16.2 (65)	42.9 (172)	
SOGS ≥ 3	30.5 (40)	16.1 (18)	53.4 (57)	
*Risky alcohol consumption* ^b^				< 0.001
Yes	42.9 (260)	15.6 (95)	41.5 (244)	
No/missing	55.1 (1399)	19.0 (471)	25.9 (608)	

Data n = 3077 18–74-year-old past-year gamblers, frequencies unweighted, percentages are weighted; statistical significance is determined by Pearson’s chi-squared test

^a^South Oaks Gambling Screen

^b^AUDIT-C, the Alcohol Use Disorders Identification Test, score for risk consumption ≥ 5 among women and ≥ 6 among men

Among those who gambled more rarely than monthly, the proportion of land-based gamblers was the highest. On the other hand, among those who gambled daily or several times a week, the proportion on multimode gamblers was the highest. A similar tendency was also seen regarding the number of game types gambled, gambling expenditure, and the number of gambling settings: the most active gambling participation was associated with multimode gambling and the least active with land-based gambling.

Gambling severity and risky alcohol consumption were significantly associated with gambling modes. Of those who had a past-year gambling problem, 30.5 % were land-based gamblers, 16.1 % were online gamblers, and more than half (53.4 %) were multimode gamblers. Of those who had used alcohol at a risk level during the past year, 42.9 % were land-based gamblers, 15.6 % were online gamblers, and 41.5 % were multimode gamblers. At the same time, among those who did not experience any gambling problems or risky alcohol consumption, land-based gambling was clearly the most common mode of gambling.

### Simultaneously Analyzed Factors and Gambling Mode

Factors reflecting socio-demographics, gambling participation, and addictive behaviors were analyzed using multinomial regression analysis (Table 2). Land-based gambling was used as the reference group for both past-year online and multimode gambling.

Male gender (OR 1.48), age between 18 and 54 (OR 1.88), and high income (Q3–Q4; OR 1.87) were significantly associated with online gambling. The odds of online gambling were significantly higher among those who gambled at least monthly (OR 1.34) and among those who spent the most money on gambling (Q2–Q4; OR 3.62). Ages between 18 and 54 (OR 2.31) and high income (Q3–Q4; OR 1.51) were significantly associated with multimode gambling. The odds of multimode gambling were also significantly higher among those who gambled at least four different game types (OR 2.96), spent the most money on gambling (Q2–Q4; OR 4.56), and gambled in at least three different gambling settings (OR 5.59). Variables measuring addictive behaviors were not significantly associated with either online or multimode gambling.

**Table 2:** Simultaneously analyzed factors: socio-demographics, gambling participation and addictive behavior associated with gambling mode

	Online gambling		Multimode gambling	
	OR	95 % CI	OR	95 % CI
*Socio-demographics*				
Man	***1.478	1.195–1.828	1.154	0.932–1.429
18–54 years	***1.884	1.505–2.359	***2.312	1.831–2.918
High net income (Q3 or Q4)	***1.874	1.506–2.332	**1.513	1.224–1.871
*Gambling participation*				
Monthly or more often	**1.340	1.062–1.692	1.053	0.836–1.326
≥ 4 game types	1.095	0.825–1.454	***2.960	2.331–3.757
High gambling expenditure (Q2–Q4)	***3.617	2.709–4.828	***4.555	3.359–6.177
Three or more gambling spaces	0.838	0.562–1.250	***5.584	4.208–7.411
*Addictive behavior*				
Gambling problems^a^	1.294	0.710–2.358	0.993	0.581–1.696
Risky alcohol consumption^b^	0.858	0.653–1.125	1.153	0.900–1.478

Reference group for online gambling (n = 566) and multimode gambling (n = 852): Land-based gambling (n = 1659); The data (n = 3077) were weighted based on gender, age and residency; Multivariate-adjusted multinomial logistic regression analysis

^a^SOGS, South Oaks Gambling Screen score ≥ 3

^b^AUDIT-C, the Alcohol Use Disorders Identification Test, score for risk consumption ≥ 5 among women and ≥ 6 among men

*< 0.05, **< 0.01, ***< 0.001

### Supplementary Analysis

**Fig. 1:**
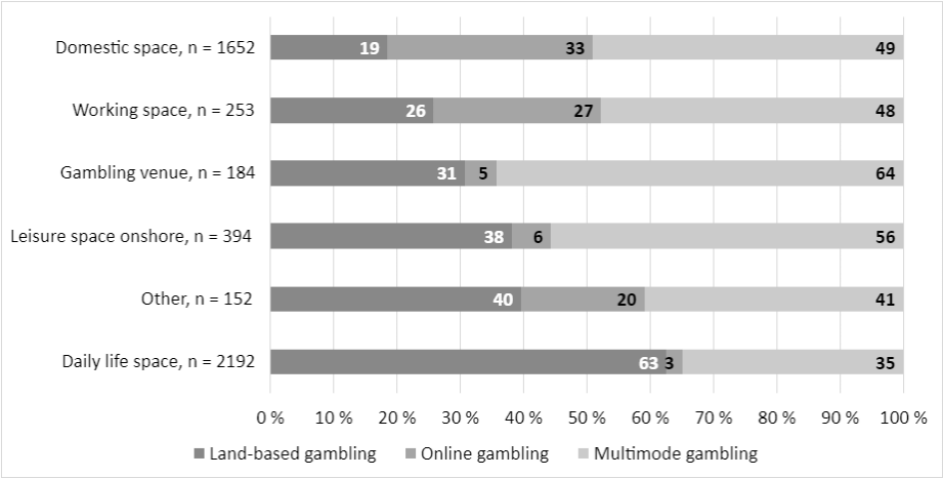
Gambling spaces and places associated with gambling mode

The model was supplemented with more detailed information about gambling settings. All gambling settings were significantly (p < 0.001) associated with gambling modes (Fig. 1). The proportions of land-based gamblers were the highest among those gambling in daily life spaces, leisure spaces onshore, and other spaces. Daily life spaces include grocery stores or malls, kiosks, and gas stations, while leisure spaces onshore include restaurants and cafes. In the Finnish context these locations are highly characterized by EGM gambling. Other spaces refer to, for example, transportation, summer cottage, sports events, vacation abroad, or a friend’s house. The proportions of online gamblers were the highest among those gambling in domestic and work spaces. The proportions of multimode gamblers were the highest among those gambling in gambling venues and leisure spaces onshore. Gambling venues refer to both Casino Helsinki and gambling arcades.

**Fig. 2:**
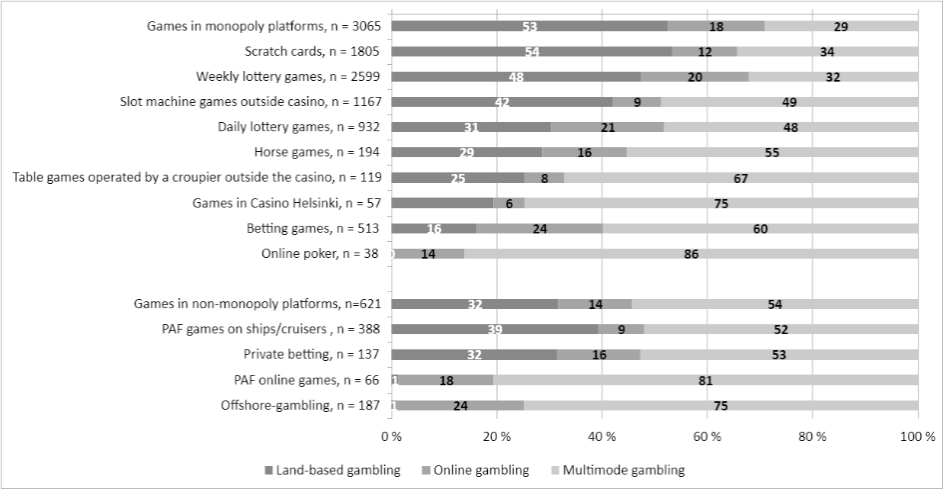
Game types gambled associated with gambling mode

Overall, more than half (53 %) of those gamblers who gambled on the monopoly platform had gambled only in land-based environments (Fig. 2). All game types were significantly (p < 0.001) associated with gambling modes. In the monopoly platform, the proportion of land-based gamblers was the highest among those gambling on scratch cards, weekly lottery games, and EGMs outside the casino. The proportion of online gamblers was the highest among those gambling on betting games, daily lottery games, and weekly lottery games. The proportion of multimode gamblers was the highest among those playing online poker, games in Casino Helsinki, and table games operated by a croupier outside the casino. On the other hand, 54 % of those gambling outside the monopoly platform were multimode gamblers. The proportion of multimode gamblers was the highest among those playing PAF games online or other offshore online games. It is noteworthy that almost all gamblers outside the monopoly platform had also gambled on the monopoly platform (98.1 %).

## Discussion

This paper has investigated how land-based, online, and multimode gambling differ in terms of socio-demographics, gambling participation, gambling settings, and addictive behaviors. The results confirm that the three modes have specific characteristics that may also be related to their harm potential. This observation highlights the importance of considering the different modes of gambling also as separate public health issues (also Mora-Salgueiro et al. 2021).

Our model showed that in the Finnish population study sample, the male gender was linked to online gambling, but not to multimode gambling. This finding diverges from previous results from Finland and elsewhere (Wardle et al. 2011; Salonen et al. 2018) that have connected the male gender also to multimode gambling. It is possible that in recent years, with overall increases in online gambling (e.g., Veikkaus 2021), female gamblers have increasingly moved online and to multimode gambling.

Our results also link younger age and higher income with both online and multimode gambling. This finding is in line with previous studies. Both online and multimode gambling have been linked to younger age also in other contexts (e.g., Gainsbury et al. 2015; Wardle et al. 2011; Pallesen et al. 2021). Gamblers in older age groups are still mostly gambling in land-based environments. Even though many land-based gambling products can also be found online, older gamblers may prefer land-based environments since gambling online requires an internet connection, adequate devices, and technological knowledge (cf. Pallesen et al. 2021). Convenience gambling settings have also been argued to cause less “environmental stress” to older gamblers than online environments where the information load is high (Finlay et al. 2006). Furthermore, older gamblers have more free time and also gamble more as a means to socialize (Tse et al. 2012). Younger generations are more familiar with online environments and interactive technology, and therefore possibly more attracted to online gambling (Pallesen et al. 2021; Hume and Sullivan Mort 2011).

Based on our results, frequent gambling was linked to the online mode, whereas versatile gambling habits (measured in terms of the number of gambling types engaged in) were linked to multimode gambling. Similar findings have been reported elsewhere (Pallesen et al. 2021). In our study, high expenditure was linked to both online and multimode gambling. Online gambling, and particularly mobile gambling, is characterized by constant availability and ease of access, enabling impulsive usage (Hume and Sullivan Mort 2011). This may explain the high overall frequency and spending particularly in comparison to land-based gambling. Multimode gambling, on the other hand, appears to be characterized by an overall versatility of gambling participation in terms of channels, gambling products, and gambling settings (cf. Salonen et al. 2018).

Surprisingly, despite the high participation and involvement in gambling among multimode gamblers, this does not appear to be reflected in gambling harms in the Finnish context. This is particularly surprising, as previous studies have connected multimode gambling to higher problem gambling prevalence (Wardle et al. 2011; Gainsbury et al. 2015; Blaszczynski et al. 2016) and online environments more generally to higher addictivity due to issues such as availability, anonymity, ease of access, and speed (Pallesen et al. 2021; Sulkunen et al. 2019). It is possible that our finding is less related to the multimode environment and more a result of the peculiarities of the Finnish gambling context. The Finnish gambling landscape is characterized by a high prevalence of land-based EGMs in convenience locations. EGMs and casino games make up over half of the Veikkaus gross gambling revenue (Nikkinen and Marionneau 2020). EGM gambling in Finland and elsewhere is also connected to elevated gambling harms (e.g., Sulkunen et al. 2019). The particularly harmful nature of land-based gambling in the Finnish context may therefore, at least partly, overshadow the harms of online and multimode gambling.

### Limitations

The Finnish Gambling population study is characterized by some statistical shortcomings. The response rate was lower in the youngest than in the oldest age groups (Salonen et al. 2020a). Unlike most surveys, men participated more actively than women in almost all age groups. It is possible that gambling is a topic that motivates men to respond. In 2019, the response rate dropped in all age groups compared to 2015, most clearly among those aged 55 to 64. The distribution of the response rate was also typical in geographical terms: the rate was lower for respondents living in cities than for those living in rural areas. According to the previous Finnish Gambling study from 2015, a lower socio-economic status was associated with a lower response rate (Kontto et al. 2020). It is possible that a similar tendency may have also caused a bias in our examination of the gambling behavior of socio-economically vulnerable individuals in the 2019 sample.

In addition to contextual factors related to the Finnish gambling field, the fact that gambling severity was not associated with the mode of gambling after controlling for other predictors may also relate to other factors. First, our study examined main effects only. It is possible that the association between gambling severity and the mode of gambling depends on the values of a third variable (i.e., interaction). This may for example be the case of the gambling participation variable. Second, a small number of participants in some sub-groups, such as problem gamblers, may be the reason for non-significant findings although the corresponding OR implies an association between the response and a covariate. These issues should be further explored in future studies.

The current study also leaves a few additional gaps that should be addressed in future research. First, we only addressed gambling and alcohol related harms. Further studies should assess whether smoking status or other addictive behaviors are connected to different gambling modes (cf. Wardle et al. 2011). Second, further studies should also address the question of how gambling modes are connected to other health and wellbeing issues. As online gambling may be connected to poorer mental health at least among men (Edgren et al. 2017), it is possible that multimode gamblers also have a different profile in terms of health. Third, more research would be needed on the effects of different types of online gambling (computer-based or mobile-based) on gambling behaviors and harms.

## Conclusion

Overall, socio-demographics and gambling participation, including gambling settings, were indicators of gambling modes. Compared to land-based gamblers, online gamblers were more often men. Land-based gamblers were older and had a lower income level than online and multimode gamblers. Furthermore, online gamblers differed based on their gambling intensity: they gambled more frequently and spent more money on gambling compared to land-based gamblers. Multimode gamblers gambled more frequently than land-based gamblers, but also in a more versatile way. Multimode gamblers participated in several types of games and gambled in several types of settings. Land-based gamblers were over-represented in daily life spaces, online gamblers in domestic and work spaces, and multimode gamblers in gambling venues and leisure spaces onshore.

## Data Availability

The Finnish Gambling 2019 population survey data is openly available for research purposes from the Finnish Social Science Data Archive (https://www.fsd.uta.fi/en/).

## References

[CR1] Abbott M, Romild U, Volberg R (2018). The prevalence, incidence, and gender and age-specific incidence of problem gambling: results of the Swedish longitudinal gambling study (Swelogs). Addiction.

[CR3] Baggio S, Dupuis M, Berchtold A, Spilka S, Simon O, Studer J (2017). Is gambling involvement a confounding variable for the relationship between Internet gambling and gambling problem severity?. Computers in Human Behavior.

[CR4] Blaszczynski A, Russell A, Gainsbury S, Hing N (2016). Mental health and online, land-based and mixed gamblers. Journal of Gambling Studies.

[CR5] Binde P, Romild U (2020). Risk of problem gambling among occupational groups: A population and registry study. Nordic Studies on Alcohol and Drugs.

[CR7] Castrén S, Perhoniemi R, Kontto J, Alho H, Salonen A (2017). Association between gambling harms and game types: Finnish population study. International Gambling Studies.

[CR6] Castrén S, Heiskanen M, Salonen AH (2018). Trends in gambling participation and gambling severity among Finnish men and women: cross-sectional population surveys in 2007, 2010 and 2015. BMJ open.

[CR8] Dowling N, Smith D, Thomas T (2005). Electronic gaming machines: are they the ‘crack-cocaine’ of gambling?. Addiction.

[CR9] Edgren R, Castrén S, Alho H, Salonen A (2017). Gender comparison of online and land-based gamblers from a nationally representative sample: Does gambling online pose elevated risk?. Computers in Human Behavior.

[CR10] Finlay K, Kanetkar V, Londerville J, Marmurek HHC (2006). The physical and psychological measurement of gambling environments. Environment and Behavior.

[CR11] Gainsbury SM (2015). Online gambling addiction: The relationship between internet gambling and disordered gambling. Current Addiction Reports.

[CR12] Gainsbury SM, Russell A, Blaszczynski A, Hing N (2015). The interaction between gambling activities and modes of access: A comparison of Internet-only, land-based only, and mixed-mode gamblers. Addictive Behaviors.

[CR13] Gainsbury SM, Angus DJ, Blaszczynski A (2019). Isolating the impact of specific gambling activities and modes on problem gambling and psychological distress in internet gamblers. BMC Public Health.

[CR14] González-Roz A, Fernández-Hermida JR, Weidberg S, Martínez-Loredo V, Secades-Villa R (2017). Prevalence of problem gambling among adolescents: A comparison across modes of access, gambling activities, and levels of severity. Journal of Gambling Studies.

[CR15] Hume M, Sullivan Mort G (2011). Fun, friend, or foe: Youth perceptions and definitions of online gambling. Social Marketing Quarterly.

[CR2] Käypä hoito (2018). Alkoholiongelmaisen hoito: Suomalaisen Lääkäriseuran Duodecimin ja Suomen Kardiologisen Seuran asettama työryhmä. Helsinki: Suomalainen Lääkäriseura Duodecim, 2018 (Treatment of alcohol problems). http://www.kaypahoito.fi

[CR16] King DL, Russell A, Hing N (2020). Adolescent land-based and internet gambling: Australian and international prevalence rates and measurement issues. Current Addiction Reports.

[CR17] Kontto J, Tolonen H, Salonen AH (2020). What are we missing? The profile of non-respondents in the Finnish Gambling 2015 survey. Scandinavian Journal of Public Health.

[CR18] Lesieur, H. R., & Blume, S. B. (1987). The South Oaks Gambling Screen (SOGS): A new instrument for the identification of pathological gamblers. *American Journal of Psychiatry*, 144(9), 10.1176/AJP.144.9.1184. 10.1176/ajp.144.9.1184 3631315

[CR19] Lesieur HR, Blume SB (1993). Revising the South Oaks Gambling Screen in different settings. Journal of Gambling Studies.

[CR20] Livingstone, C., Rintoul, A., de Lacy-Vawdon, C., Borland, R., Dietze, P., Jenkinson, R., … & Hill, P. (2019). Identifying effective policy interventions to prevent gambling-related harm. Melbourne: Victorian Responsible Gambling Foundation. https://responsiblegambling.vic.gov.au/resources/publications/identifying-effective-policy-interventions-toprevent-gambling-related-harm-640.

[CR21] Mora-Salgueiro J, García-Estela A, Hogg B, Angarita-Osorio N, Amann BL, Carlbring P, Colom F (2021). The prevalence and clinical and sociodemographic factors of problem online gambling: A systematic review. Journal of Gambling Studies.

[CR22] Nikkinen J, Marionneau V (2020). On the efficiency of Nordic state-controlled gambling companies. Nordic Studies on Alcohol and Drugs.

[CR23] Pallesen S, Mentzoni RA, Morken AM, Engebo J, Kaur P, Erevik EK (2021). Changes Over Time and Predictors of Online Gambling in Three Norwegian Population Studies 2013–2019. Frontiers in Psychiatry.

[CR24] Raybould JN, Larkin M, Tunney RJ (2021). Is there a health inequality in gambling related harms? A systematic review. BMC Public Health.

[CR26] Salonen AH, Hellman M, Latvala T, Castrén S (2018). Gambling participation, gambling habits, gambling-related harm, and opinions on gambling advertising in Finland in 2016. Nordic Studies on Alcohol and Drugs.

[CR25] Salonen, A., Hagfors, H., Lind, K., & Kontto, J. (2020a). Gambling and problem gambling: Finnish Gambling 2019: Prevalence of at-risk gambling has decreased. THL Statistical report 9/2020a.

[CR27] Salonen, A., Lind, K., Hagfors, H., Castrén, S., & Kontto, J. (2020b). Rahapelaaminen, peliongelmat ja rahapelaamiseen liittyvät asenteet ja mielipiteet vuosina 2007–2019: Suomalaisten rahapelaaminen 2019. (Gambling, gambling problems, and attitudes towards gambling 2007–2019. Finnish gambling 2019). https://www.julkari.fi/handle/10024/140820.

[CR28] Sulkunen, P., Babor, T. F., Ornberg, J. C., Egerer, M., Hellman, M., Livingstone, C., … & Rossow, I. (2019). *Setting limits: Gambling, science and public policy*. Oxford University Press 10.1111/add.15241 33084199

[CR29] Svensson J, Romild U, Nordenmark M, Månsdotter A (2011). Gendered gambling domains and changes in Sweden. International Gambling Studies.

[CR30] Tobias-Webb J, Griggs RL, Kaufman N, Clark L (2019). Role reversal: The influence of slot machine gambling on subsequent alcohol consumption. Journal of Gambling Studies.

[CR31] Tse S, Hong S-I, Wang C-W, Cunningham-Williams R (2012). Gambling behavior and problems among older adults: A systematic review of empirical studies. Psychological Sciences and Social Sciences.

[CR32] Veikkaus (2021). Vuosi- ja vastuullisuusraportti 2020. (Annual and responsibility report 2020) https://cms.veikkaus.fi/site/binaries/content/assets/dokumentit/vuosikertomus/2020/vuosi_-ja-vastuullisuusraportti_2020.pdf.

[CR33] Wardle H, Moody A, Griffiths M, Orford J, Volberg R (2011). Defining the online gambler and patterns of behaviour integration: Evidence from the British Gambling Prevalence Survey 2010. International Gambling Studies.

[CR34] Williams, R. J., Volberg, R. A., & Stevens, R. M. (2012). *The population prevalence of problem gambling: Methodological infuences, standardized rates, jurisdictional diferences, and worldwide trends*. Ontario Problem Gambling Research Centre

